# Avoiding rash decisions about zoster vaccination: insights from cost-effectiveness evidence

**DOI:** 10.1186/s12916-018-1231-3

**Published:** 2018-12-18

**Authors:** Chester B. Good, Natasha Parekh, Inmaculada Hernandez

**Affiliations:** 1 Center for Value Based Pharmacy Initiatives, University of Pittsburgh School of Medicine Health Plan, Pittsburgh, 15219 PA USA; 2 University of Pittsburgh School of Medicine, Pittsburgh, PA 15213 USA; 3 University of Pittsburgh School of Pharmacy, Pittsburgh, PA 15213 USA

**Keywords:** Vaccine, Immunization, Herpes zoster, Cost-effectiveness, Decision-making

## Abstract

de Boer and colleagues present a cost-effectiveness analysis based in the Netherlands of two vaccines available for the prevention of herpes zoster. Zostavax® was the first vaccine available for the prevention of herpes zoster in older adults. A live-attenuated vaccine, Zostavax is not free of limitations, which include a relatively low efficacy that wanes over time and its contraindication among immunocompromised individuals. The recently available adjuvanted herpes zoster subunit vaccine Shingrix® overcomes some of these limitations. The herpes zoster subunit vaccine is more efficacious than Zostavax, and it can be administered to immunosuppressed individuals. However, the herpes zoster subunit vaccine is considerably costlier and requires a booster injection. In order to clarify the value of each vaccine, de Boer and colleagues compare the cost-effectiveness of no vaccination, and of vaccination with Zostavax or the herpes zoster subunit vaccine in four cohorts of older adults from the perspective of the Netherlands. Whereas neither vaccine was cost-effective under the willingness-to-pay threshold of €20,000 per quality-adjusted life year, the authors find the herpes zoster subunit vaccine to be cost-effective in some scenarios under a €50,000 per quality-adjusted life year threshold.

Please see related article: https://bmcmedicine.biomedcentral.com/articles/10.1186/s12916-018-1213-5

In this issue, de Boer and colleagues [1] present a cost-effectiveness analysis of two vaccines available for the prevention of herpes zoster (HZ), the live-attenuated Zostavax® vaccine (ZVL) and the adjuvanted HZ subunit vaccine (Hz/su) Shingrix®, from the perspective of the Netherlands.

## The live-attenuated Zostavax® vaccine

Released in 2006, ZVL was the first vaccine available for the prevention of HZ and post-herpetic neuralgia (PHN) in older adults [2, 3]. ZVL is a live-attenuated vaccine that addressed a huge unmet medical need for millions of individuals at risk of secondary infection of latent varicella zoster virus. The incidence of HZ has been estimated at 3.6 and 5.2 cases per 1,000 person-years in the U.S. and the Netherlands, respectively [1, 4]. Most HZ cases occur in patients over the age of 50 years, and the incidence of the infection increases with age [4]. One-third of patients over 70 years of age who develop HZ experience PHN, and 10% of patients experience one or more non-pain complication [4].

The ZVL has notable limitations. First, in the Shingles Prevention Study (SPS), the ZVL only lowered the incidence of HZ by 51% and the risk of PHN by 67% [5]. Importantly, the vaccine was significantly less effective in participants over 70 years, and its efficacy was found to wane over time with no significant decrease in HZ incidence after 8 years [6]. Second, because it is a live-attenuated vaccine, ZVL was contraindicated in immunocompromised individuals [2, 3] who have a higher risk for developing HZ and severe HZ-related complications. Moreover, the prospective safety study of the SPS found a higher incidence of severe adverse events with ZVL than expected [5, 7]. As a result, the U.S. Food and Drug Administration mandated a follow-up study to assess the safety of ZVL [8], which found that ZVL was not associated with an increased risk of serious adverse events [9].

## The adjuvanted Shingrix® vaccine

Given the concerns regarding the ZVL, uptake was lower than expected. After 7 years on the market in the U.S., coverage with the ZVL in patients over 60 years was estimated at 19.5% [10]. In this context, the approval of a newer zoster vaccine in 2017 was welcomed. Shingrix is an adjuvanted HZ subunit vaccine (HZ/su) that overcomes many of the limitations of the ZVL. First, the HZ/su has significantly greater efficacy than the ZVL [11]. In particular, among patients over 70 years the efficacy of the HZ/su has been estimated at 91% compared to 38% for the ZVL [5, 12]. Second, the HZ/su is not contraindicated in patients with immunocompromised states. Finally, the HZ/su is indicated in patients over the age of 50 (as opposed to 60 years for the ZVL).

While the HZ/su has significant advantages over the ZVL, it has three main limitations. Compared with placebo, those that received the HZ/su in clinical trials experienced a higher risk of adverse events, including grade 3 systemic reactions [11]. These side effects are particularly concerning because they may discourage patients from obtaining the required booster injection within 2–6 months of the original vaccination. Of note, prior studies of vaccines that require a second shot indicate that adults frequently do not get the entire series. For example, only 43.6% and 61.1% of patients over 65 years receive the second hepatitis A and B immunizations, respectively, within 1 year of receiving the initial shots [13]. The ZVL does not require a second injection; however, re-vaccination at 10 years has been studied to overcome its lack of efficacy over time [14]. Additionally, it remains unknown whether the effectiveness of the HZ/su will decline as rapidly as does that of the ZVL, although it is expected that the immune response to the HZ/su will be better maintained owing to its superior initial antibody response. Finally, the HZ/su is costlier than the ZVL.

## Cost-effectiveness of Zostavax® and Shingrix®

Given the higher effectiveness of the HZ/su, but the lower price, greater tolerability, and lack of need for a booster injection with the ZVL, a comprehensive comparison of downstream patient outcomes and the costs associated with each vaccine was necessary to inform payers, providers, and patients on choosing between these alternatives. Within this context, de Boer and colleagues [1] present an elegant cost-effectiveness analysis of the HZ/su, the ZVL with or without a 10-year booster, and no vaccination, in four cohorts of 50, 60, 70, and 80 years of age, from the perspective of the Netherlands. Specifically, they express their findings as the maximum cost under which the ZVL or HZ/su would be cost-effective compared to no vaccination under willingness-to-pay thresholds of €20,000 per quality-adjusted life year (QALY) and €50,000 per QALY. Input estimates for the incidence of HZ and PHN and associated costs were obtained from Dutch national registries, and health disutilities were extracted from a Dutch prospective study. The authors performed several sensitivity analyses to evaluate the robustness of their results for variations in assumptions and in input parameters.

As expected, the authors found that the HZ/su prevents significantly more cases of HZ than the ZVL, resulting in a higher number of QALYs. For the HZ/su, the number needed to vaccinate to avoid one case of HZ was less than 11 for all age cohorts, whereas for the ZVL it was 22.8, 34.9, and 117.0 for patients of 60, 70, and 80 years of age, respectively. Vaccination with the HZ/su was most cost-effective in the 70-year-old cohort, and the cost-effectiveness of the ZVL was highest for the 60-year-old cohort. Specifically, for the 60-year-old cohort, the HZ/su would be cost-effective compared to no vaccination for costs below €104 (per series), and the ZVL would be cost-effective if priced at €51.40 or below. For the 70-year-old cohort, the HZ/su would be cost-effective compared to no vaccination for costs below €109, and the ZVL would be cost-effective if priced at €27.50 or below. Given the current prices of both vaccines in the Netherlands, neither vaccine was cost-effective in any patient population under the €20,000 per QALY willingness-to-pay threshold. However, many countries or healthcare systems use a higher willingness-to-pay threshold. Under the €50,000 per QALY threshold, the HZ/su vaccine would be cost-effective in some scenarios.

There are several important assumptions in their analysis. First, the base case analysis assumes 100% compliance with both doses of the HZ/su, which is unlikely in real-world clinical practice. To relax this assumption, the authors performed sensitivity analyses in which compliance for the second dose was 90%, 70%, and 50%. Under the 50% compliance scenario, the HZ/su is cost-effective compared to no vaccination in the 70-year-old cohort if priced around €30 less than in the base case scenario. This suggests that the impact of missing the second dose on the cost-effectiveness of the vaccine is considerable, which should support efforts by health systems to improve compliance with the booster injection. Second, because the waning effectiveness rate of the HZ/su is unknown, the authors assumed a 4.1% per year decline over a time horizon of 15 years in patients 70 years or older. Using lower waning rates significantly improved the cost-effectiveness of the vaccine.

## Conclusions

We commend the authors for their thorough and robust cost-effectiveness analysis. This evidence will be extremely useful for healthcare systems hoping to model the impact of coverage decisions regarding vaccination to prevent HZ. Additionally, their estimates will provide a benchmark for manufacturers to lower prices sufficiently to approach reasonable cost-effectiveness estimates. Finally, the evidence on the varying cost-effectiveness of each vaccine for different age groups will guide decision-makers in identifying patients who will benefit most from vaccination.

## Abbreviations

HZ: Herpes zoster
HZ/su: Adjuvanted herpes zoster subunit vaccine
PHN: Post-herpetic neuralgia
SPS: Shingles Prevention Study
ZVL: Live-attenuated Zostavax® vaccine

## References

[1] De Boer PT, van Lier A, de Melker H, van Wijck AJM, Wilschut JC, van Hoek AJ, et al. Cost-effectiveness of vaccination of immunocompetent older adults against herpes zoster in the Netherlands: a comparison between the adjuvanted subunit and live-attenuated vaccine. BMC Med. 2018; 10.1186/s12916-018-1213–5. Accessed 11 Dec 2018. PMC6282315 30518427

[2] U.S. Department of Health and Human Services. What everyone should know about Zostavax. 2018. https://www.cdc.gov/vaccines/vpd/shingles/public/zostavax/index.html. Accessed 15 Nov 2018.

[3] European Medicines Agency. Zostavax. 2018. https://www.ema.europa.eu/medicines/human/EPAR/zostavax#authorisation-details-section. Accessed 15 Nov 2018.

[4] Yawn BP, Saddier P, Wollan PC, St Sauver JL, Kurland MJ, Sy LS (2007). A population-based study of the incidence and complication rates of herpes zoster before zoster vaccine introduction. Mayo Clin Proc..

[5] Oxman MN, Levin MJ, Johnson GR (2005). A vaccine to prevent herpes zoster and postherpetic neuralgia in older adults. N Engl J Med..

[6] Morrison VA, Johnson GR, Schmader KE (2015). Long-term persistence of zoster vaccine efficacy. Clin Infect Dis..

[7] Good CB (2007). Varicella-zoster vaccine. N Engl J Med..

[8] Woo EJ, Ball R, Barun MM (2007). Varicella-zoster vaccine. N Engl J Med..

[9] Schmader KE, Levin MJ, Gnann JW, McNeil SA, Vesikari T, Betts RF (2012). Efficacy, safety, and tolerability of herpes zoster vaccine in persons aged 50–59 years. Clin Infect Dis..

[10] Zhang D, Johnson K, Newransky C, Acosta CJ (2017). Herpes zoster vaccine coverage in older adults in the U.S., 2007–2013. Am J Prev Med..

[11] Lal H, Cunningham AL, Godeaux O, Chilbek R, Diez-Domingo J, Hwang SJ (2015). Efficacy of an adjuvanted herpes zoster subunit vaccine in older adults. N Engl J Med..

[12] Cunningham AL, Lal H, Chlibek KR (2016). Efficacy of the herpes zoster subunit vaccine in adults 70 years of age and older. N Engl J Med..

[13] Nelson JC, Bittner RC, Bounds L, Zhoa S, Baggs J, Donahue JG (2009). Compliance with multiple-dose vaccine schedules among older children, adolescents, and adults: results from a vaccine safety datalink study. Am J Public Health..

[14] Weinberg A, Popmihajlov Z, Schmader KE, Johnson MJ, Caldas Y, Salazar A, et al. Persistence of varicella-zoster virus cell-mediated immunity after the administration of a second dose of liver herpes zoster vaccine. J Infect Dis. 2018; 10.1093/infdis/JIY514. Accessed 11 Dec 2018. 30165651

